# Modeling Uncertainty for the Double Standard Model Using a Fuzzy Inference System

**DOI:** 10.3389/frobt.2018.00031

**Published:** 2018-03-28

**Authors:** Noelia Torres, Leonardo Trujillo, Yazmin Maldonado

**Affiliations:** Departamento de Ingenieria Electrica y Electronica, Instituto Tecnologico de Tijuana, Tijuana, Mexico

**Keywords:** ambulances, emergency medical services, bases, double standard model, triangular fuzzy set, fuzzy inference system

## Abstract

This paper studies the issue of uncertainty in the ambulance location problem to cover the maximum number of demand points in a city. The work is based on the double standard model (DSM), a popular coverage model where two radii are considered to cover a percentage of the demand points twice. Uncertainty is introduced in the expected travel time between an ambulance and a demand point, before computing the optimal placement of ambulances in potential bases by solving the linear program posed by the DSM. The following three approaches are considered: (1) solving the DSM without uncertainty; (2) uncertainty in the travel time is based on triangular fuzzy set; and (3) a fuzzy inference system (FIS) with a rule base derived from the problem properties, which is the main contribution of this work. Results show that considering uncertainty can have a significant effect on the solutions for the DSM, with the solutions produced with the FIS approach achieving a higher total coverage of the demand. In conclusion, the proposed strategy could provide a reliable and effective tool to support decision making in the ambulance location problem by considering uncertainty in the ambulance travel times.

## Introduction

In recent years, the development of computational support systems for emergency medical services (EMS) has attracted a growing amount of attention from researchers. In EMSs, a crucial factor that must be considered is response time. For example, let us consider cardiac arrest, the American Heart Association concluded that in the first 4–6 min after a person suffers a cardiac arrest she (he) can begin to suffer from permanent brain damage or even brain death. However, normal heart rhythm can be restored if advanced life support (ALS) is provided early. Studies have shown that the probability of a patient surviving is reduced by 7–10% with each passing minute in which defibrillation and ALS are not provided, and resuscitation is mostly unsuccessful after 10 min (EMSWorld.com, 2014).

Another example is traffic accident, where the number of deaths was estimated to be 1.25 million in 2013. Half of all fatalities caused by traffic accidents are pedestrians, cyclists, and motorcyclists. Death rates from such accidents are considerably higher in developing countries (OMG, 2015). Therefore, appropriate and timely response times to such incidents are indispensable in highly urban areas.

In 2013, the Red Cross of Tijuana (RCT) covered about 98% of the EMS requests (Cruz Roja de Tijuana, 2012), providing medical attention to 37,000 emergency calls. It does this with 13 ambulances that are distributed in 8 bases that cover a population of approximately 1.6 million people in an area of 1,243 km^2^ (INEGI, 2010). This means that, on average, each ambulance serves about 123,000 people and each base must cover an average of 155 km^2^. This contrasts, for instance, with the US, where by the 1990s there was about 1 ambulance per 51,000 inhabitants (Braun et al., 1990).

This work is motivated by the lack of resources and their sub-optimal use, which is evident when we consider that the average response time of RCT ambulances was approximately 14 min with an SD of 7 min. Such performance is unsatisfactory, and there is a pressing need to optimize the use of all available resources.

In this work, the main objective is to study the ambulance location problem that seeks to determine where to place the available ambulances within a city. To do so, we use a well-known coverage model and solve it with linear programming. However, our contribution is to consider uncertainty in the estimated travel time between an ambulance and a possible demand point. In particular, we explore the use of fuzzy sets and a fuzzy inference system (FIS), which provide a natural way to describe uncertainty in a human readable form. The results show that the coverage provided by the solutions found can be substantially different, when uncertainty is explicitly considered.

This paper is organized as follows. Section “Background” presents a review of the state of the art. Section “Double Standard Model” describes the double standard model (DSM), which is the coverage model used in this work. Section “Travel Time Estimation for Ambulances” presents how uncertainty in the travel time is modeled in our work, with our main contribution being the use of an FIS. Section “Experiments and Results” presents the description of the experiments and obtained results. Finally, Section “Conclusion and Future Work” outlines the conclusion and describes future work.

## Background

Traditionally, the ambulance location problem deals with two types of decisions: (a) which sites in a city should be used as bases, and (b) determining how many ambulances should be placed in each base. What follows will deal mostly with models for this problem, while solution methods will not be covered in depth. Suffice it to say that many solution methods have been applied, including linear programming (MatLab, 2015) (which is also used in this work), Tabu search (Gendrau et al., 1997; Luke, 2014), evolutionary algorithms (Jones, 2002; Eiben and Smith, 2015), and Fuzzy logic (Zadeh, 2015).

Models for this problem can be divided into three main groups (Li et al., 2011): (1) covering models that focus on locating ambulances such that the demand can be covered within a certain amount of time; (2) p-median models that focus on minimizing the total (or average) distance between ambulance and demand points; and (3) p-center models that minimize the maximum distance between ambulances and demand points. Among the three groups, the coverage models are the most frequent and therefore reviewed next. These models are concerned with maximizing demand coverage, considering that a demand point is covered if it can be reached within a predefined time or distance by an ambulance, referred to as a coverage radius.

According to the literature, the first coverage model was the location set coverage problem (Toregas et al., 1971). It considers mandatory coverage, the goal is to determine the minimum amount of resources required so that all demand points are covered. Another well-known model is the maximum coverage location problem (Church and ReVelle, 1974), where given a limited number of resources the goal is to maximize the demand covered. Both of these models produce solutions where a demand point can be left uncovered once an ambulance response to a service. In the literature, there are two general proposals to overcome this drawback. The first is to provide multiple coverage, such as the DSM (Gendrau et al., 1997), the goal of which is to achieve full coverage within a large radius and maximize double coverage within a shorter radius. A second approach is to model the problem probabilistically, as in the maximum expected cover location problem and the maximum availability location problem.

The DSM considers a static number of ambulances, while a dynamic version has also been developed (Laporte et al., 2009). The multi-capacities ambulance location model was proposed in Shiah et al. (2009), which concurrently considers three different types of capacities: travel distance, populations, and the location EMS calls.

In the study by Schmid (2012), it was shown that solving the static location problem using fixed travel times may not suffice, due to the speed variations of the ambulances and because coverage areas change throughout the day. The problem was solved by the authors using a variable neighborhood search. Fuzzy logic as a solution method was used by Davari et al. (2011), who proposed the fuzzy maximum coverage location problem, which is solved by simulated annealing. Another fuzzy covering model is studied in the study by Rezaei and Zarandi (2011). The reduction of ambulance response time is studied using a hybrid approach in the study by Zarkeshzadeh et al. (2016). The method considers the rate of incoming emergency calls, available resources, the probability of hospitalization of patients, as well as the distances and locations of the emergency units.

As previous work on this topic, in 2017 we have reported on modeling the demand for EMS in Tijuana, Baja California, Mexico, followed by the optimization of the location of ambulances for the RCT. We used data from more than 10,000 emergency calls from 2013 to model and classify the demand for EMS in different scenarios that provide different perspectives of the demand throughout the city, considering factors such as the time of day or whether the incident occurred in a work day or an off-day (Dibene et al., 2017). In that work, the DSM was extended to generate robust solutions that could generalize to different scenarios. A total of 1,000 possible bases are considered and the solution was generated with integer linear programming. Results showed how the solutions found could improve coverage relative to the approach taken by the RCT at the time without requiring additional resources.

## Double Standard Model

In the DSM, the objective function computes the demand covered twice within small radius (*r*_1_) time units. Below, we describe DSM problem and parameters, defined as follows.

Maximize:

(1) $$f(x)=\sum_{i\in l}d_{i}x_{i2}$$

Subject to:

(2) $$\sum_{j\in J}\delta_{ij}y_{j}\geq 1\;\forall i\in I$$

(3) $$\sum_{i\in I}d_{i}x_{i1}\alpha \sum_{i\in I}d_{i}$$

(4) $$\sum_{j\in I}\gamma_{ij}y_{j}\geq \;\;x_{i1}+x_{i2}\;\forall i\in I$$

(5) $$x_{i2}\leq x_{i1}\;\forall i\in I$$

(6) $$\sum_{j\in J}\gamma_{ij}=p$$

(7) $$y_{j}\leq p_{j}\;\forall j\in J$$

(8) $$\sum_{i\in I}\delta_{ij}z_{ij}\leq w_{j}y_{ij}\;\forall j\in J$$

(9) $$\sum_{j\in J}\delta_{ij}z_{ij}\leq d_{i}\;\forall i\in I$$

(10) $$y_{j}\geq 0\;\forall j\in J$$

(11) $$z_{ij}\geq 0\;\forall i\in I,\;\forall j\in J$$

(12) $$x_{i,k}\in \{0,1\}\;\forall i\in I,k\;\in \{1,2\}$$

(13) $$y_{j}\in \mathbb{Z}\;\forall j\in J$$

(14) $$Z_{ij}\in \mathbb{Z}\;\forall i\in I,\;\forall j\in J$$

where *i* ∈ *I*: demand point; *j* ∈ *J*: potential ambulance location; *r*_1_: small radius; *r*_2_: large radius; *p*: total the available ambulances; *t_ij_*: the distance between the point of demand and the base, this variable is the travel time and is calculated using three models, described in the following section; α: percentage of total demand which must be covered by an ambulance located within *r_1_* radius; *d_i_*: total demand at patient location *i*; *p_j_*: the largest number of ambulances at the ambulance location; *w_j_*: the capacity of each ambulance when it is placed in ambulance location *j*; $\delta_{i,j}=\{\begin{array}{cc} 1 & t_{ij}\leq r_{2}\\ 0 & o.w \end{array},$ if the distance between the point of demand and the base is less or equal to r_2_, all demands are covered within the large radius; $\gamma_{i,j}=\{\begin{array}{cc} 1 & t_{ij}\leq r_{2}\\ 0 & o.w \end{array},$ if the distance between the demand point and the base is less than or equal to r_1_, all demands are covered within the small radius; Variables: *y_j_* = the number of ambulance located at the vehicle location *j*. *x_i_*_1_ = 1, if the place *i* is covered by one ambulance through the small radius. *x_i_*_2_ = 11, if the place *i* is covered by two or more ambulance through the small radius. *z_ij_* = current demand at the patient location *i* which is covered by one ambulance at the ambulance location *j*.

The goal of the objective function (1) is to maximize the demand covered by at least two ambulances within the small radius. Constraint (2) ensures that one ambulance covers each demand point within the larger radius *r*_2_. Constraint (3) states that a fraction *α* of the total demand must be covered within the small radius. The left-hand side of constraint (4) has the number of ambulances covering demand point *i* within *r*_2_, and the right-hand side is equal to 1 if the demand point *i* is covered exactly once within the small radius and equal to 2 if it is covered at least twice. Constraint (5) states that a demand point *i* cannot be covered twice if it is not covered once. Constraints (4) and (5) state that if two ambulances are within *r*_1_, the demand point is said to be covered twice. Constraint (6) states that all ambulances must be assigned to a base. Constraint (7) verifies the maximum number of ambulances at each base *j*. Constraint (8) ensures that the demand covered by base *j* depends on the ambulance locations. Constraint (9) states that the total demand in demand point *i* must be covered within the large radius *r*_2_. Constraints (10) and (11) express that some variables cannot be negative and constraint (12) states that the coverage index is a binary variable. Constraint (13) shows that the total available ambulances is an integer. Finally, constraint (14) states that *z_ij_* must be an integer.

## Travel Time Estimation for Ambulances

**Figure 1:**
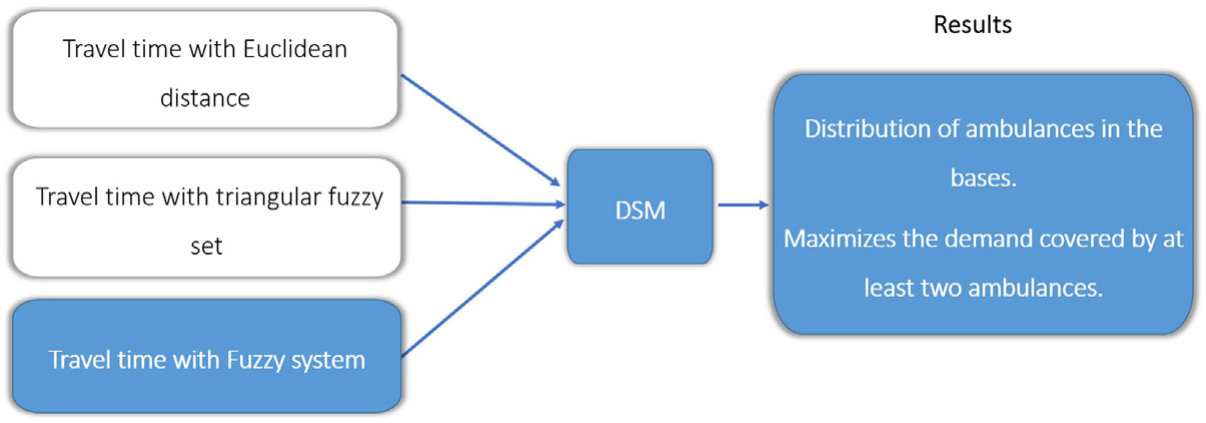
Different methods used to estimate the ambulance travel times for the double standard model (DSM).

The EMS providers prefer to assign the closest, in terms of travel time, available ambulance to respond to a new emergency. Thus, it is vital to have accurate estimates of the travel time of each ambulance to the emergency location. Therefore, travel times play a central role in positioning the ambulance bases (Laporte et al., 2009). The intuition behind this work is that if the variability in travel times is accounted for explicitly then EMS management and planning can be significantly improved. To show this, we use three different methods to calculate the ambulance travel time: (1) using fixed travel times (Laporte et al., 2009); (2) introducing uncertainty in the travel time using a triangular fuzzy set (TFS) as proposed by Lahijanian et al. (2016); and the main proposal in this work, (3) using an FIS to account for uncertainty in the travel time between an ambulance and a demand point. A simulation is setup to consider travel times in a road network, following the study by Lahijanian et al. (2016). Random data are generated in a hypothetical area of 900 km^2^, using 30 bases and 100 random demand points. Figure 1 shows the three versions of the DSM.

### Basic DSM Approach

To calculate the travel time between a demand point and an ambulance, we used the Euclidian distance divided by speed, using three different speeds (40, 60, and 80 km/h) for the ambulance.

### DSM With TFS

The DSM with a TFS considers the uncertainty for the travel time between locations of the patients and the locations of the ambulances, based on the study by Lahijanian et al. (2016).

**Figure 2:**
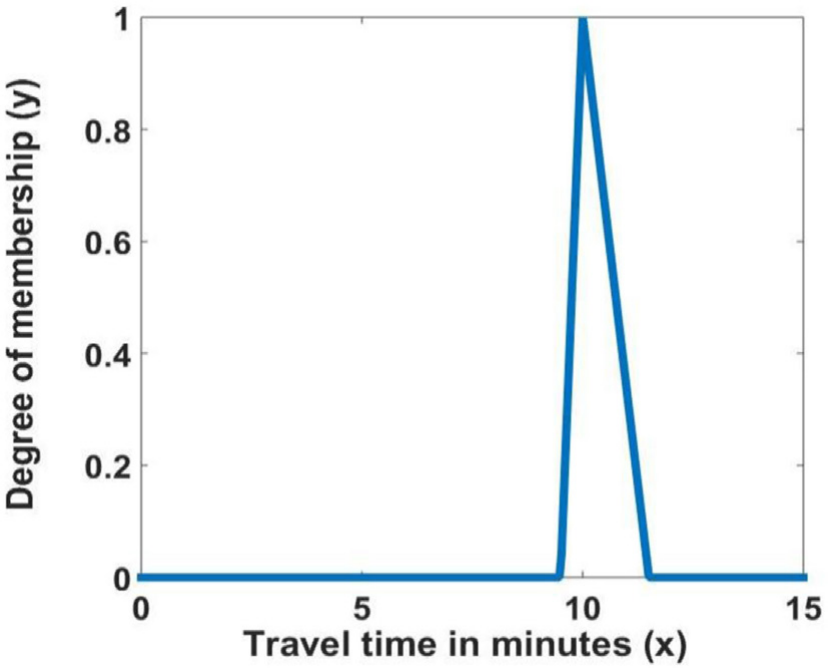
Travel time membership function.

1. The first step is to convert the time to a triangular fuzzy number, adding uncertainty in the trip.
2. The TFS, shown in Figure 2, is defined as a function of *x*, where *y* depends on three scalar parameters *a, b*, and *c*, defined as:
  (15) $$\mu (x)=\{\begin{array}{ccc} \frac{x-a}{b-a} & \text{if} & a\leq x\leq b\\ \frac{x-c}{b-c} & \text{if} & b\leq x\leq c\\ 0 & & \text{Otherwise} \end{array}$$
3. The defuzzification is done using the centroid method to obtain a crisp number, this is done by extracting the average from the triangular fuzzy number.

Notice that if the fuzzy set is symmetric, then the estimated travel time will be the same as the original value. This method is only useful if the Fuzzy set is asymmetric. If it is asymmetric to the right (*c* − *b* > *b* − *a*) then the estimated travel time is larger than the original time and *vice versa*. In our case, we set *b* equal to the input travel time, *a* = 0.95 × *b* and *c* = 1.15 × *b*. In this formulation, the estimated travel time is always an over estimation of the original travel time given by time and distance. In what follows, we consider a more robust formulation of uncertainty using an FIS.

### DSM With FIS

**Figure 3:**
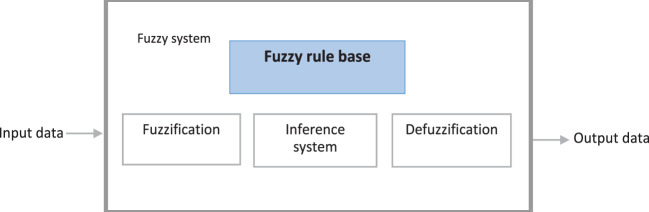
Fuzzy inference system.

This is the main contribution of this paper, where a Mamdani FIS is used to determine the travel time between the demand point and the ambulance. The basic FIS system (Zadeh, 2015; Grande et al., 2017; Paramo-Carranza et al., 2017; Rubio, 2017) is composed as shown in Figure 3.

**Figure 4:**
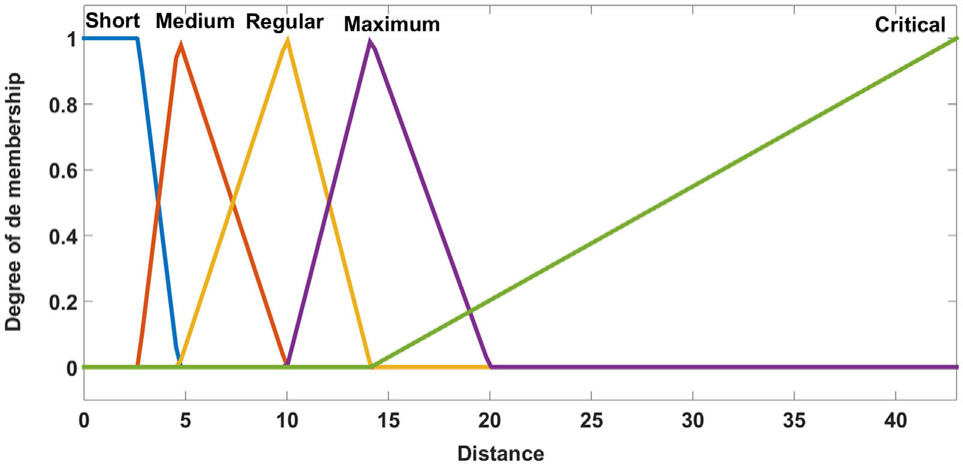
Membership functions of distance.

**Figure 5:**
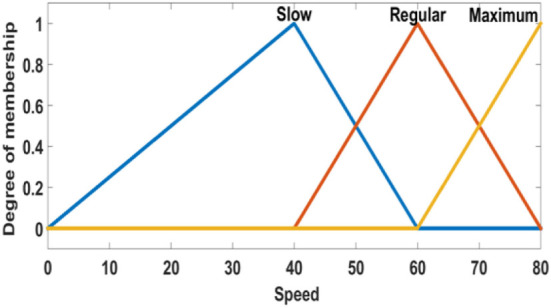
Membership functions of speed.

**Figure 6:**
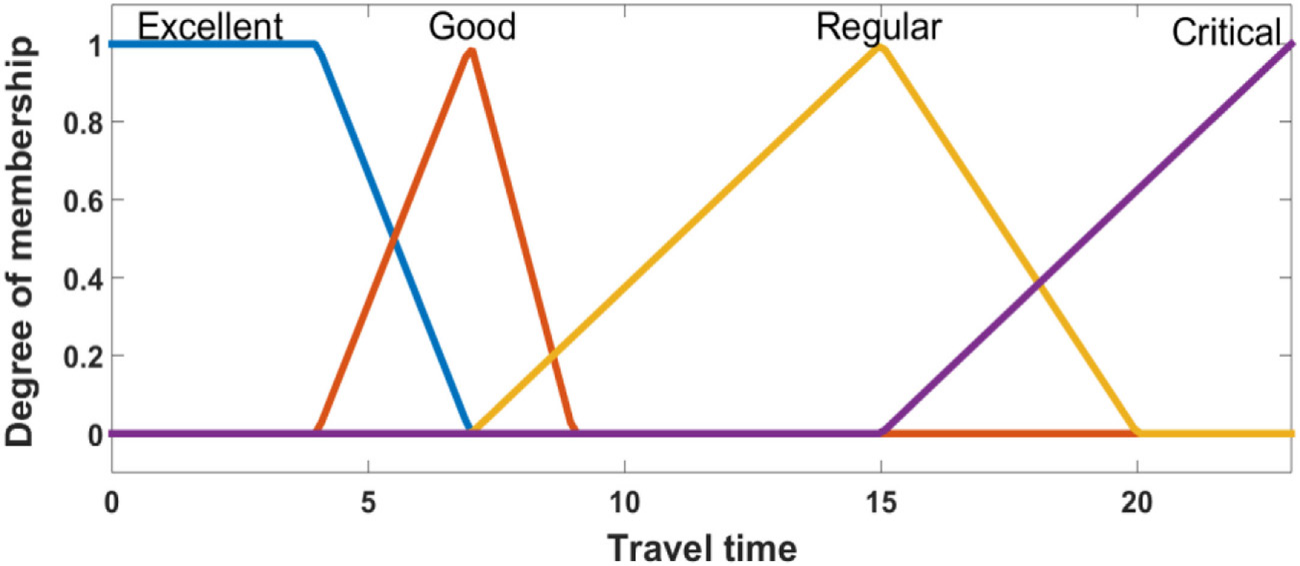
Membership functions of travel time.

Two inputs are considered for the FIS. First is the distance between the demand point and the ambulance, and the second is given by the speed of the ambulance. The output of the FIS is the travel time. The linguistic values are shown in Figures 4–6.

In the fuzzification stage, the data are transformed into linguistic terms (fuzzy sets). The inference system simulates the human reasoning process by making fuzzy inference on the inputs with IF-THEN rules, and the defuzzification transforms the fuzzy set into a crisp value.

Fifteen fuzzy rules are proposed, and the centroid defuzzification method is used to obtain the estimated travel time. Table 1 shows the fuzzy rules used. We have four consequents and their interpretation is:

- *Excellent*: when the demand point is covered in a radius of 4 min.
- *Good*: when the demand point is covered in a radius of 7 min.
- *Regular*: when the demand point is covered in a radius of 15 min.
- *Critical*: when the demand point is out of coverage.

**Table 1:** Matrix of inference rules used with the proposed fuzzy inference system.

		Input speed		

		Slow	Regular	Maximum
Input distance	Short	Good	Excellent	Excellent
	Mean	Good	Good	Excellent
	Regular	Regular	Good	Good
	Maximum	Critical	Regular	Good
	Review	Critical	Critical	Critical

## Experiments and Results

For this work, randomly generated demand points and ambulance locations are used. We use as reference the study by Lahijanian et al. (2016), a square province is assumed with a dimension of 30 km × 30 km.

A total of *n* = *100* demand points are randomly placed using a continuous uniform distribution within the considered area. Then, *m* = 30 points, which represents that the possible bases are placed in the following manner. The square is divided into a 3 × 3 grid, and it is assumed that more possible ambulance based will be found in the middle of the province (downtown), generating *2m/10* potentials bases in this zone, and *m/10* potential bases are generated in each of the eight remaining locations in the grid. Experiments and results are divided into three subsections, which are described below.

### Experiment 1: Comparison of the Three Methods Used to Estimate Ambulance Travel Times

The first group of experiments will try to provide a broad overview of the effect that each method has on the solutions found in the DSM linear program. We will try to get a general characterization by considering different numbers of available ambulances and different top speeds. The following parameters for the DSM are considered:

- *r*_1_: 7 min (small radius).
- *r*_2_: 15 min (large radius).
- *p*: [10,15,20, 25, and 30], total available ambulances.
- α: 0.65; percentage of total demand which must be covered by an ambulance located within radius *r*_1_, this parameter is based on the tests carried out in the study by Lahijanian et al. (2016).

To compare the performance of the three methods, we generated 100 different configurations, considering:

- Random distribution of demand points.
- Random distribution of bases.
- Maximum number of ambulances in each base set randomly in the range [1–3].
- Different speeds (40, 60, and 80 km/h).

**Figure 7:**
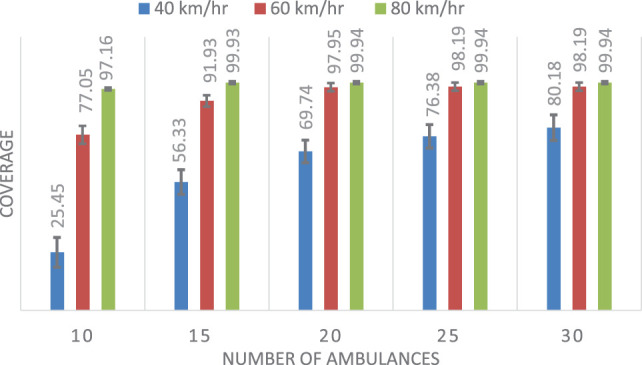
Mean and SD of the coverage using double standard model.

**Figure 8:**
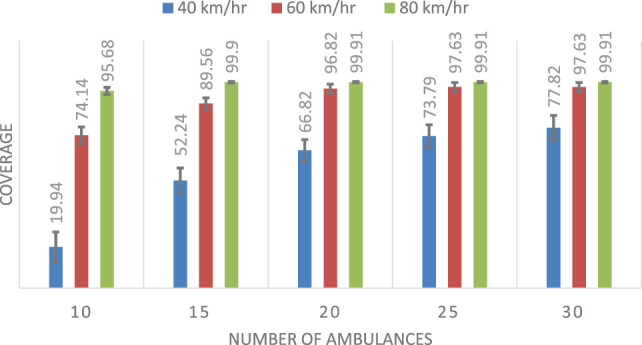
Mean and SD of the coverage using double standard model with triangular fuzzy set.

**Figure 9:**
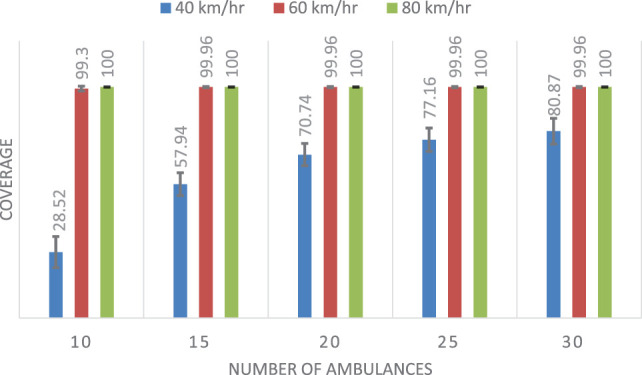
Mean and SD of the coverage using double standard model with fuzzy inference system.

Table 2 shows the mean and SD of the coverage results for each of the three methods considering different speeds. Figures 7–9 show the same comparison showing the mean coverage and SD with error bars, by solving the 100 different problems considering different speeds. Analyzing the results, we can see, in all the cases with different speeds, DSM with FIS method is the best option, the solution is due to the FIS is robust with uncertainty information. These results show that the best results are obtained when using the FIS to estimate the travel times. Moreover, these results seem to be quite consistent, independent of the number of available ambulances and the speed of the ambulances.

**Table 2:** Comparison results for the coverage of the three methods, showing the mean (μ) and SD.

Number of ambulances	Coverage (μ ± SD) (40 km/h)			Coverage (μ ± SD) (60 km/h)			Coverage (μ ± SD) (80 km/h)		
	DSM	DSM TFS	DSM FIS	DSM	DSM TFS	DSM FIS	DSM	DSM TFS	DSM FIS
10	25.45 ± 6.54	19.94 ± 7.28	**28.52 ± 6.74**	77.05 ± 3.92	74.14 ± 4.04	**99.30 ± 1.03**	97.16 ± 1.70	95.68 ± 2.09	**100 ± 0.00**
15	56.33 ± 5.39	52.24 ± 6.07	**57.94 ± 4.93**	91.93 ± 2.48	89.56 ± 2.73	**99.96 ± 0.24**	99.93 ± 0.33	99.90 ± 0.41	**100 ± 0.00**
20	69.74 ± 4.95	66.82 ± 5.22	**70.74 ± 4.83**	97.95 ± 1.80	96.82 ± 2.18	**99.96 ± 0.24**	99.94 ± 0.31	99.91 ± 0.40	**100 ± 0.00**
25	76.38 ± 5.15	73.79 ± 5.46	**77.16 ± 5.06**	98.19 ± 1.79	97.63 ± 2.16	**99.96 ± 0.24**	99.94 ± 0. 31	99.91 ± 0.40	**100 ± 0.00**
30	80.18 ± 5.62	77.82 ± 5.96	**80.87 ± 5.60**	98.19 ± 1.79	97.63 ± 2.16	**99.96 ± 0.24**	99.94 ± 0.31	99.91 ± 0.40	**100 ± 0.00**

*Bold values are the best values of the experiment*.

### Experiment 2: Comparison in a Typical Scenario

In this case, we are interested in analyzing a more typical scenario. We repeat the experiments described in Experiment 1, with the following two modifications. First, the maximum number of ambulances allowed in each base is set to *p_j_* = 2. Second, the speed of the ambulance is set to 60 km/h. These settings are consistent with those suggested by the RCT.

**Figure 10:**
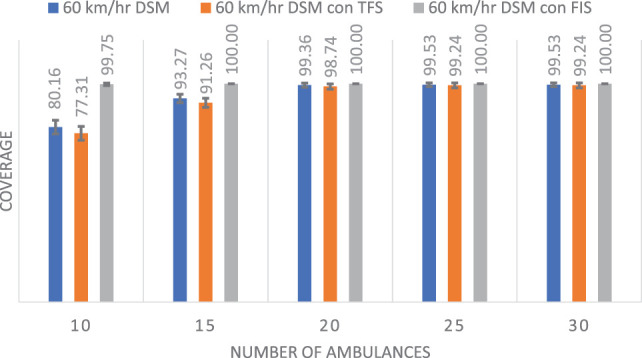
Comparison of coverage (mean and SD) with a speed of 60 km/h.

Figure 10 shows the average results of 100 randomly generated problems and the SD shown with error bars. Results are clear, the DSM with the FIS method achieves the best results. Notice that with 10 ambulances, the coverage is 99.75% for this approach, while for 15, 20, 25, and 30 ambulances the coverage is 100%.

**Figure 11:**
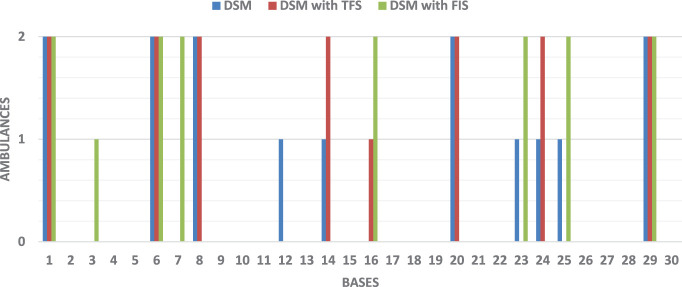
Comparison of number of ambulances located in each base with 15 available ambulances.

To compare the differences between each method, we take a single case as an example. We consider a total of 15 available ambulances. Figure 11 shows how the number of ambulances (vertical axis) located in each base (horizontal axis). In this case, double coverage with the standard DSM is 93%, 91% for the DSM using a TFS, and 100% for the DSM using a FIS to account for uncertainty in travel time.

### Experiment 3: Analysis of the DSM With FIS

**Figure 12:**
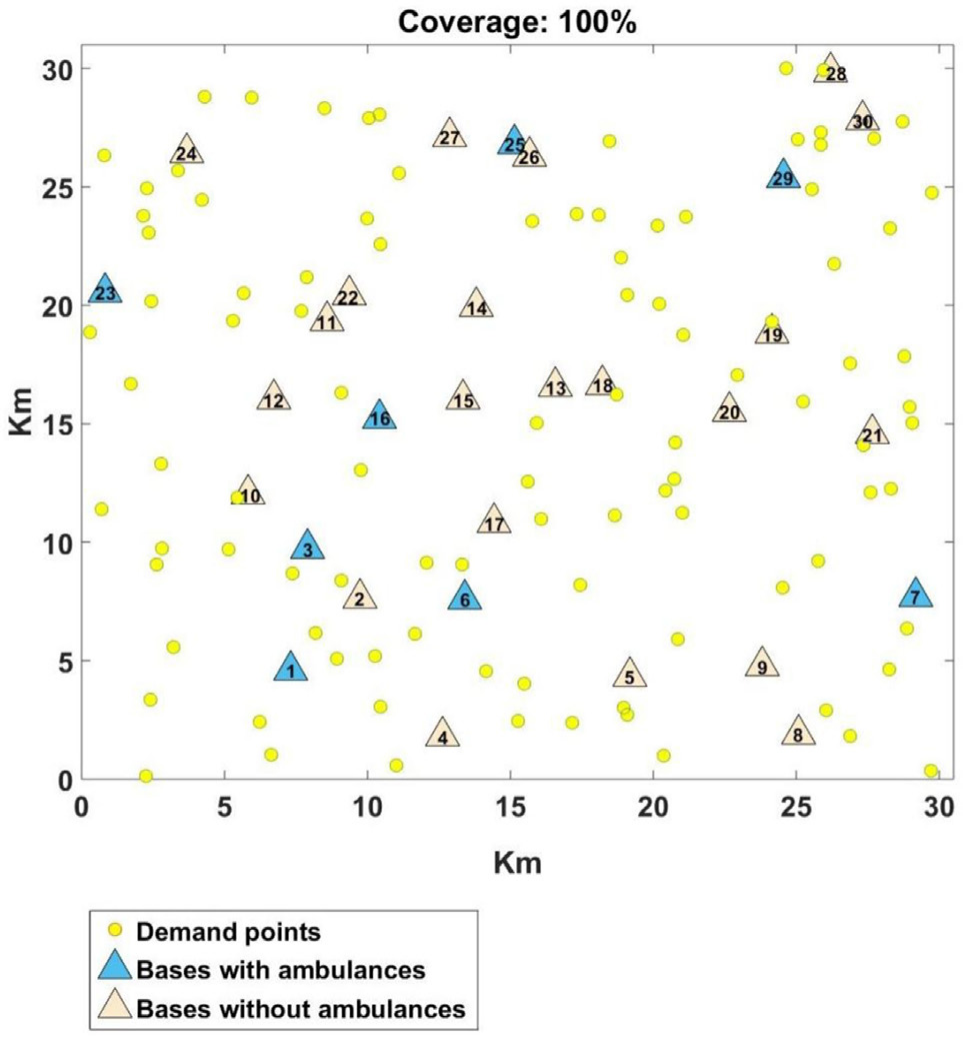
Distribution of ambulances found by the double standard model with fuzzy inference system.

Let us now provide a more detailed discussion of the result achieved for the DSM with the FIS. Figure 12 shows the problem used for the comparison in Figure 11, where the DSM with FIS achieved 100% coverage. The triangles represent the 30 bases and the circles indicate the 100 demand points. The distribution of ambulances, as also seen in Figure 11, is: Base 1, Base 6, Base 7, Base 16, Base 23, Base 25, and Base 29 with 2 ambulances and Base 3 with 1 ambulance (a total of 15 ambulances).

**Figure 13:**
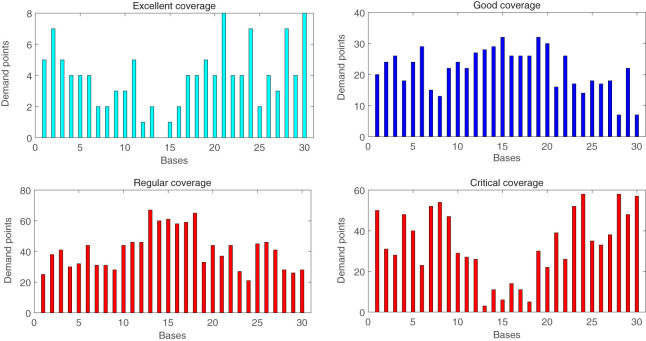
Number of demand points covered by each base, considering different possible fuzzy consequents (excellent, good, regular, and critical).

Considering the consequents of the fuzzy rules used by the FIS, Figure 13 shows the number of demand points that are covered by each base in a time that is considered to be excellent, good, regular, or critical, as defined in the Section “DSM with FIS.”

**Figure 14:**
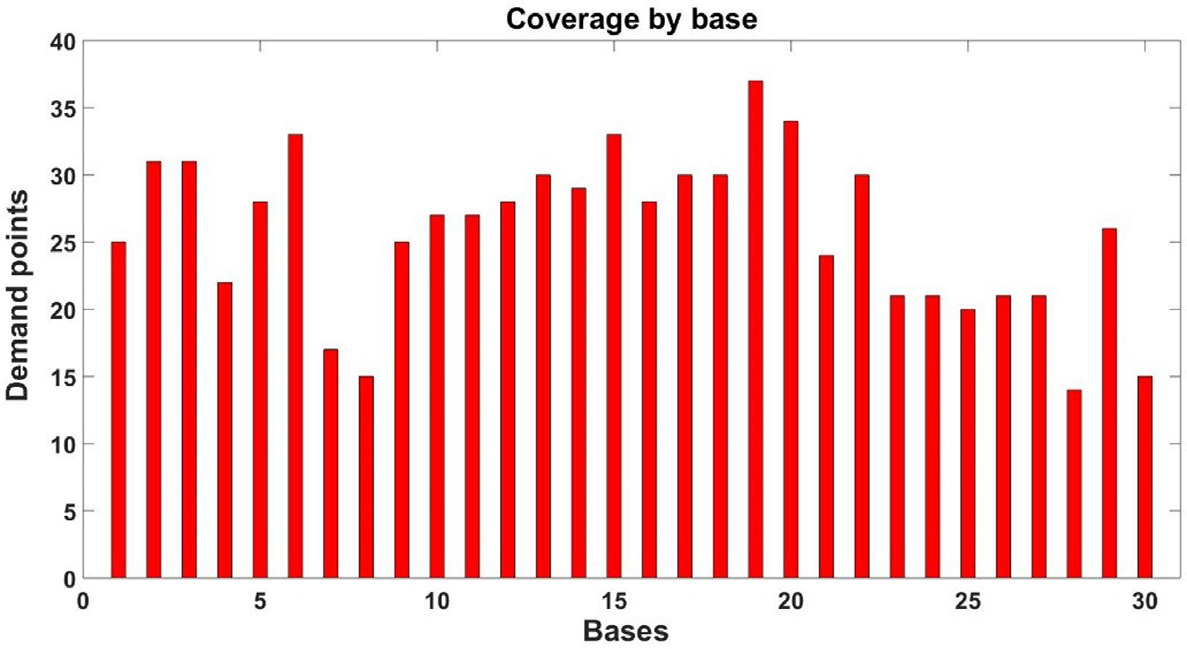
Demand points that cover each base.

**Figure 15:**
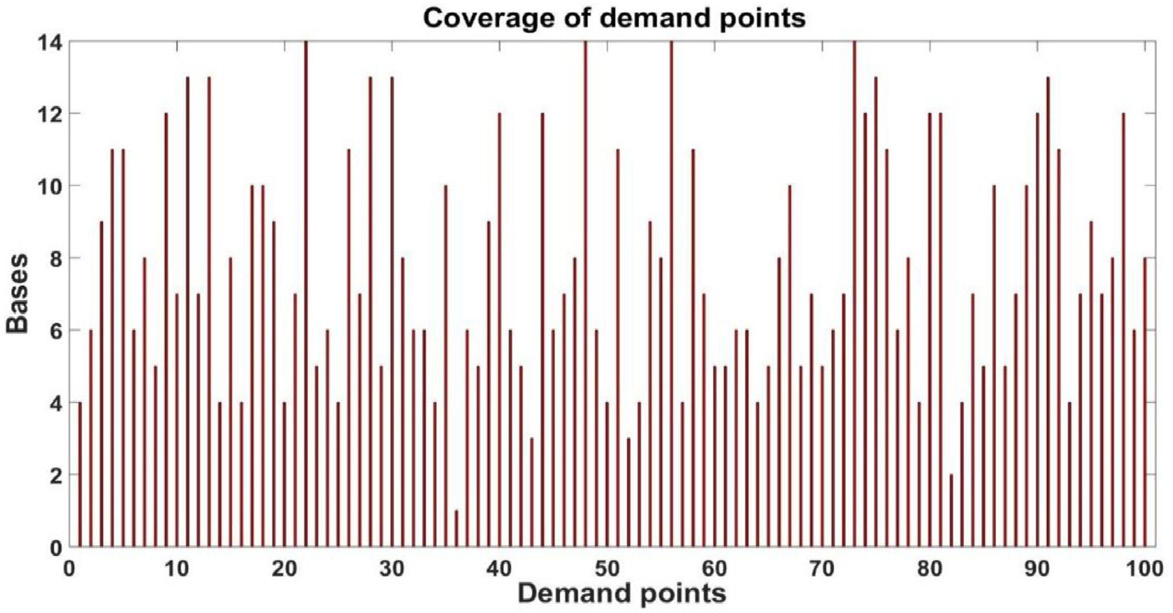
Number of bases covering demand points.

Now, considering the small radius in the DSM, we can provide a more detailed analysis of the total coverage achieved. Figure 14 shows the number of demand points covered by each base within the small radius. Notice that all bases cover no less than 10 demand points, with some based covering as much as 30 demand points. Conversely, Figure 15 shows the number of bases that provide coverage to each demand point within the small radius.

## Conclusion and Future Work

In this work, the uncertainty is introduced into in the estimated travel time between a demand point and a candidate base for the ambulance location problem solved with the DSM. Three methods are compared, using no uncertainty, modeling uncertainty with a TFS, and a more complete approach that used an FIS. The coverage obtained with the DSM improves when uncertainty is considered, particularly using the FIS which achieves 100% coverage in cases where the standard approach does not. These results clearly indicate the importance of considering uncertainty in this real-world domain.

In future work, we will focus on applying the proposed method on real data taken from the EMS of the RCT. The application of the model will improve the location of ambulances and the response time to emergency services will be timely, a most important problem in a city where resources are scarce.

## Author Contributions

NT studied and implemented the algorithms to solve the ambulance location problem and implemented the fuzzy approach to travel time uncertainty. YM and LT were responsible for analyzing the results obtained.

## Conflict of Interest Statement

The authors declare that the research was conducted in the absence of any commercial or financial relationships that could be construed as a potential conflict of interest.
